# Dried Blood Spot Analysis for Simultaneous Quantification of Antiepileptic Drugs Using Liquid Chromatography–Tandem Mass Spectrometry

**DOI:** 10.1002/rcm.10064

**Published:** 2025-05-12

**Authors:** Mariam M. Abady, Ji‐Seon Jeong, Ha‐Jeong Kwon

**Affiliations:** ^1^ Organic Metrology Group, Division of Chemical and Material Metrology Korea Research Institute of Standards and Science Daejeon Republic of Korea; ^2^ Department of Bio‐Analytical Science University of Science and Technology Daejeon Republic of Korea; ^3^ Department of Nutrition and Food Science National Research Centre Cairo Egypt

**Keywords:** antiepileptic drugs, dried blood spots, LC–MS/MS, method validation, punch techniques, therapeutic drug monitoring

## Abstract

**Rationale:**

Dried blood spot (DBS) sampling for the therapeutic drug monitoring of antiepileptic drugs offers practical advantages, including minimal invasiveness and ease of collection. However, for precise therapeutic management, its accuracy and reliability in quantification need to be validated.

**Methods:**

This study validates DBS sampling for the analysis of 11 antiepileptic drugs using liquid chromatography‐tandem mass spectrometry (LC‐MS/MS), overcoming the physicochemical challenges associated with DBS samples.

**Results:**

The acetonitrile‐based DBS extraction method demonstrated high efficiency for the 11 antiepileptic drugs. Accuracy and precision within 6% were achieved in both intra‐ and inter‐day assays, with good selectivity, minimal matrix effects, and negligible carryover. All antiepileptic drugs exhibited stability in DBS samples for at least 30 days at room temperature, confirming proper handling and storage of the DBS samples. A 3 mm diameter disc punched from a DBS produced accurate results for all target drugs.

**Conclusions:**

The optimized method provided a time‐ and cost‐effective solution, showing a strong correlation between drug concentrations in whole blood, thereby supporting the suitability of DBS sampling as a promising and advanced method for antiepileptic drug monitoring.

## 1 Introduction

Dried blood spot (DBS) sampling, a microsampling method, offers a promising and less invasive alternative to venipuncture [1, 2]. This method requires only a small amount of peripheral blood and can be conveniently performed by patients at home, reducing the need for plastic, offering improved stability at room temperature, and lowering biohazard risks when compared to liquid blood samples [3]. Due to such benefits, DBS sampling is widely used in various clinical fields, including qualitative testing for HIV and viral hepatitis [2, 4] and newborn screening (first pioneered in 1963 for phenylketonuria [5]). Advancements in analytical science and technology are expected to enable not only the screening and identification but also the precise quantification of biomarkers using DBS sampling. In particular, therapeutic drug monitoring (TDM), which requires frequent blood sampling, may benefit from less invasive DBS methods in improving patient compliance [1, 6, 7, 8].

Due to the limited sample volume in DBSs (5–50 μL of blood), which may lead to low concentrations of the target analytes, detection and quantification require advanced methods [9], with liquid chromatography combined with tandem mass spectrometry (LC–MS/MS) being the most representative as it offers both selectivity and sensitivity [10]. As a result, LC–MS/MS has been widely embraced in DBS‐based analysis for a diverse array of drugs, including immunosuppressants, antimicrobials, antidiabetics, and antidepressants [11, 12, 13, 14, 15, 16]. Despite its advantages, DBS sampling faces quantitative analytical challenges in clinical usage due to biases arising from differences in hematocrit levels, red blood cell/plasma ratios, and uneven distributions of the targets [17, 18], which may be solved through the continuous collection of clinical application data. To facilitate the adoption of DBS for TDM, it is essential to validate and standardize sample preparation methods, LC–MS/MS‐based analytical procedures for the quantification of a wide range of drugs, and appropriate sampling techniques, including assessments of how punching position affects accuracy [7, 9].

In our previous paper, we proposed a method for the simultaneous analysis of multiple generations of antiepileptic drugs (AEDs) in serum [19], common targets for TDM. To determine whether this method could be adapted to DBS samples and what considerations would need to be taken into account to do so, further assessment is required. In clinical laboratories, where analysis time and cost are critical factors, it is difficult to set up more complex sample preparation methods beyond extracting DBSs with a universal solvent (such as methanol (MeOH) or acetonitrile (ACN)) to simultaneously dissolve the target drugs. It is, therefore, necessary to evaluate appropriate extraction solvents and assess whether the analysis can be performed without bias in the extraction matrix.

**FIGURE 1:**
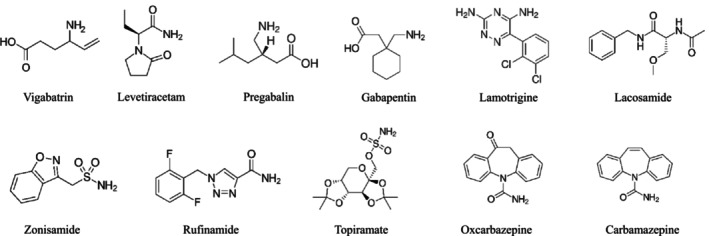
Chemical structures and names of the target antiepileptic drugs (AEDs).

Our study takes a significant step forward in making DBS sampling a viable alternative for routine TDM of AEDs in clinical laboratories by optimizing an LC–MS/MS method for the simultaneous quantification of 11 AEDs (Figure 1), a major expansion from studies analyzing fewer AEDs [7]. This work also comprehensively examines all the necessary factors for transitioning AED analysis using DBSs to clinical use. We optimized sample preparation methods, validated a robust LC–MS/MS analytical procedure including accuracy, precision, selectivity, and matrix effects, and evaluated stability under different DBS storage conditions. Furthermore, we assessed the impact of different disc sampling techniques and confirmed their consistency. By addressing these foundational aspects, our work lays a solid foundation for the reliable and practical application of DBS sampling in clinical laboratories. It should be noted, though, that to complete the clinical transition, further research is needed to determine how to correct or incorporate variations stemming from RBC/plasma ratios, capillary blood, and hematocrit characteristics, depending on the drug being analyzed. This study provides a standardized protocol that will facilitate the accumulation of data necessary for evaluating these variations and advancing the use of DBS‐based TDM for AEDs in clinical practice.

## 2 Materials and Methods

### 2.1 Reagents and Materials

Certified reference materials for pharmaceutical secondary standards of pregabalin (99.5 ± 1.0%), vigabatrin (99.4 ± 0.6%), and gabapentin (99.9 ± 0.2%) and solid‐form high‐performance liquid chromatography (HPLC)‐grade reference materials (≥ 98%) of lamotrigine, levetiracetam, rufinamide, topiramate, lacosamide, zonisamide, carbamazepine, and oxcarbazepine were purchased from Sigma‐Aldrich (St. Louis, Missouri, United States). Liquid‐form lamotrigine‐^13^C,^15^N_4_ (500 mg/L), gabapentin‐^13^C_3_ (100 mg/L), oxcarbazepine‐^13^C (100 mg/L), and levetiracetam‐D_6_ (100 mg/L) isotopes were purchased from Cerilliant (Darmstadt, Germany), and solid forms of 4‐aminocyclohexanecarboxylic acid (ACHC) and lacosamide‐D_3_ were obtained from Sigma‐Aldrich. HPLC‐grade dimethyl sulfoxide (DMSO), MeOH, and ACN were purchased from Thermo Fisher Scientific (Waltham, Massachusetts, United States). Formic acid and ammonium formate (analytical grade) were sourced from Sigma‐Aldrich. Freshly collected whole blood samples from healthy donors were acquired from the Korean Red Cross with approval under IRB protocol KRISS‐IRB‐2022‐1. The preparation of samples and standards was carried out using an XPE205 analytical balance (Mettler Toledo, Columbus, Ohio, United States) to ensure accurate gravimetric measurements. Deionized water (DW) with a conductivity of 0.054 μS/cm, essential for preparing samples, standards, and the mobile phase, was purified on‐site using a Milli‐Q Plus system from Millipore (Burlington, Massachusetts, United States).

### 2.2 Preparation of Stock and Standard Solutions

Rufinamide, topiramate, carbamazepine, and oxcarbazepine were dissolved in DMSO according to the manufacturer's instructions, while the other AEDs were dissolved in MeOH to prepare stock solutions at 1000 mg/kg by gravimetric method. The stock solutions were stored at −20°C and used within a maximum period of 1 month. Standard solution mixtures at six concentration levels were gravimetrically prepared by mixing the stock solutions of the 11 AEDs, with the concentrations adjusted by diluting with DW according to the therapeutic range of each drug.

Among the internal standards (ISs), ACHC was dissolved in MeOH and lacosamide‐D_3_ in DMSO to prepare solutions at 1 mg/g. These were then mixed with the remaining liquid‐form isotopes to prepare the final IS mixture at a concentration of 100 ng/g for ACHC, oxcarbazepine‐^13^C, and levetiracetam‐D_6_; 250 ng/g for lamotrigine‐^13^C,^15^N_4_ and lacosamide‐D_3_; and a concentration of 1 mg/g for gabapentin‐^13^C_3_. The IS mixture was combined with the six levels of standard solution mixtures in equal volumes for use as calibrators.

### 2.3 Preparation of DBS Samples

Freshly collected whole blood from healthy RH^+^ type O donors was collected in sterilized containers, and a protease inhibition cocktail (Cat. no.: B14013, Biotool, Switzerland) was added to ensure sample stability. Following 18 h of homogenization at 4°C, 0.15 mL of AED standard solution was gravimetrically added to 10 mL of whole blood to prepare four concentration levels. Two of these levels (Level 1 and Level 2) fell within the therapeutic range, while the other two levels (Level 3 and Level 4) were adjusted for linearity. Following an additional 2 h homogenization step at 4°C, a volumetric pipette was used to dispense 50 μL of the AED‐spiked blood to the center of a printed circle on filter paper (Whatman 903 level, Macherey‐Nagel, Düren, Germany). The blood was continuously mixed during the spotting process, and the spots were dried overnight at 4°C under low humidity (< 30%) in the dark [20]. After drying, they were stored at −20°C in sealed aluminum bags with desiccants until further analysis of the AEDs.

### 2.4 Preparation of Working Samples

After placing the DBS samples in a 1.5 mL Eppendorf tube, 50 μL of the IS mixture was added, followed by the addition of 250 μL of DW to mimic the original blood consistency [21]. Then, 0.7 mL of ACN, an organic solvent selected through preliminary experiments (details in Text S1), was added to extract the AEDs and induce protein precipitation while shaking at room temperature for 1 h. The extract was transferred to a 1.5 mL tube and centrifuged at 16200 *g* for 5 min, after which the supernatant was evaporated to dryness using a vacuum centrifugal evaporator at 55°C. The dried residue was reconstituted in two stages with 75 μL of MeOH and 75 μL of DW and then filtered through a 0.22 μm PVDF centrifugal filtration device and spun at 16 200 *g* for 20 min. The reconstituted sample was then transferred into autosampler vials for LC–MS/MS analysis.

### 2.5 LC–MS/MS Analysis

The LC–MS/MS instrumental conditions optimized in our previous paper [19] were applied. The chromatography conditions using a Shimadzu HPLC dual‐gradient pump and SIL‐30 AC autosampler are provided in Text S2, and the MS/MS conditions using a Triple TOF 5600 + system (SCIEX, Framingham, Massachusetts, United States) in positive electrospray ionization (ESI) mode are detailed in Table S1.

### 2.6 Method Validation

#### 2.6.1 Linearity, Limit of Detection (LOD), and Limit of Quantification (LOQ)

Six levels of calibration solutions were gravimetrically prepared with ISs and analyzed using the developed LC–MS/MS method. The procedure was performed in triplicate over three consecutive days. Calibration curves were generated by plotting the ratio of the analyte's peak area to that of the IS against the analyte‐to‐IS concentration ratio, using the regression equation *y = ax + b*, where *y* and *x* represent the regression variables. Six ISs were matched to the 11 targets as determined in our previous paper [19]. Following guidelines from the ICH (International Council for Harmonisation of Technical Requirements for Pharmaceuticals for Human Use) [22], the LOD and LOQ were derived by multiplying the standard deviation of the response (σ) divided by the slope of the calibration curve (S) by 3.3 and 10, respectively, ensuring reliable and precise quantification of the AEDs in low sample volumes, which is critical for microsampling applications.

#### 2.6.2 Selectivity and Matrix Effects

Selectivity and matrix effects were evaluated using samples extracted from blank DBSs without AEDs. First, blank DBS extracts were analyzed using LC–MS/MS, and any interference peaks in the extracted ion chromatogram (EIC) were examined to assess selectivity.

A postcolumn infusion method was then performed to evaluate the matrix effects. While blank DBS extracts were injected into LC–MS/MS, standard solutions of AEDs at LOQ levels were introduced at a flow rate of 10 μL/min through a T‐connector positioned between the analytical column and the ion source. The MS/MS signals of each AED, consistently supplied after the column, were monitored in the EIC to confirm whether they were maintained without any signal degradation under the DBS matrix. Additionally, AED standard solutions at four different concentrations were added to blank DBS extracts and analyzed in six replicates to compare the intensity with and without the DBS matrix.

#### 2.6.3 Precision and Accuracy

Intra and interday assays were performed by testing DBS samples spiked with drugs at four concentration levels, with six replicates for each level tested daily over three consecutive days. Accuracy was evaluated through the calculation of the mean relative error (%), while precision was determined using the relative standard deviation (% RSD) of the measured values.

### 2.7 Stability

The short‐ and long‐term stabilities of the AEDs in DBSs were evaluated following the storage of drug‐spiked DBS samples at four concentration levels under varying conditions: room temperature for durations of 3 days, 1 week, and 1 month, and 4°C for 1 month. Each storage condition was tested by performing the complete sample preparation, preservation, and storage as well as the analysis process six times.

### 2.8 Disc Sampling Tests

To confirm the effectiveness and applicability of the developed method to DBS sampling, different disc sampling approaches were utilized to provide a comprehensive evaluation: whole disc sampling and partial‐spot sampling including central‐punched disc (punch‐in) and peripheral‐punched disc (punch‐out) methods. Sampling for disc analysis was carried out using a standard 3.0 mm paper punch or biopsy puncher. Ten disc samples, carefully punched from spiked DBSs using the designated puncher, were positioned in a 2 mL conical‐bottom tube containing 30 μL of the IS solution. The procedures remained consistent across whole‐spot analysis and liquid blood/serum, with adjustments made in the extraction volumes based on sample volume. Four samples were gravimetrically prepared for each technique by the addition of standard solution mixtures into whole blood, followed by identical preparation, extraction, and analysis procedures as detailed earlier.

## 3 Results and Discussion

### 3.1 Sample Extraction

**FIGURE 2:**
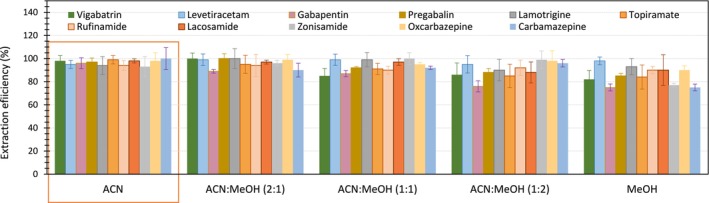
Optimization of protein precipitation using various ratios of miscible organic solvents: ACN, MeOH, and their mixtures (2:1, 1:1, and 1:2). This process was applied to dried blood spot (DBS) samples spiked with an investigational drug mixture at Level 3 (see Section 2.3). Error bars indicate the standard deviation from four replicates.

Made up of dried blood on filter paper, DBSs contain impurities from both the paper and blood sample, leading to higher matrix interference compared to serum [23]. Solid‐phase extraction and liquid–liquid extraction are effective for matrix removal; however, they often face challenges in achieving high extraction efficiency for multiple components with varying chemical properties. Additionally, their application in clinical laboratories may be limited due to cost‐ and labor‐intensive processes [19, 24, 25]. Our previous study [19] applied a relatively simple pretreatment method using a miscible organic solvent (ACN or MeOH) to induce protein precipitation and efficiently extract all AEDs from serum. To determine whether this method could efficiently extract the target AEDs from DBS samples, which have a more complex matrix than serum, the extraction efficiencies of the 11 AEDs were evaluated using ACN, MeOH, and their mixtures (Figure 2). We observed no significant difference in extraction efficiency among different organic solvent compositions for most drugs, with an average extraction efficiency of 92%. However, the average extraction efficiency of vigabatrin, zonisamide, and carbamazepine when extracted with MeOH was 78%, which is not a stable, high recovery rate compared to the extraction efficiency with ACN. We, therefore, selected ACN as the solvent for the simultaneous extraction of the drugs, adjusting its concentration to 70% of the total volume, consistent with a previous report [19]. But critically, protein precipitation with an organic solvent may cause matrix effects [26], especially in the more complicated DBS matrix. The following sections address method validation, covering the evaluation of matrix effects.

### 3.2 Method Validation

#### 3.2.1 Selectivity, Carryover, and Matrix Effects

**FIGURE 3:**
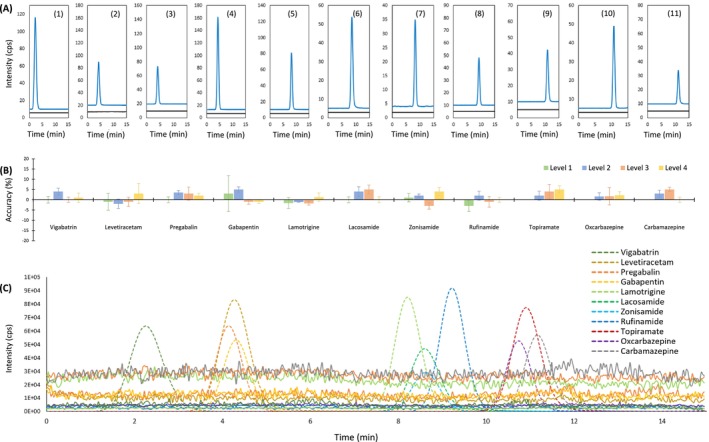
Assessment of method selectivity and matrix effects. (A) Chromatograms showing individual AEDs (blue) overlaid with purified blank DBSs (black). (B) Accuracies (% on the y‐axis) and precision (error bars) of measuring AEDs in purified blank DBSs (*n* = 6). (C) Extracted ion chromatograms (EICs) displaying post‐column infusion matrix effects obtained from the LC–MS/MS analysis of a blank DBS extract. Dotted lines represent the EICs of the 11 AEDs as references.

In TDM, the limited blood volume and intricate matrix of DBSs pose issues for achieving efficient extraction and high selectivity, as well as optimal sensitivity [27]. In this study, selectivity was assessed by examining EICs of the target drugs in purified DBS extracts. No overlapping peaks were detected at the same retention times in the EICs when analyzing purified blank DBSs, confirming high identification and separation of the target drugs with inhibited interference (Figure 3A). Moreover, there was no significant carryover observed in the EICs of blank DBSs after injecting the highest level of calibrator (Figure S1). Additionally, matrix effects were quantitatively assessed by comparing the signals of the same concentrations of AEDs in standard solutions and blank DBS extracts; results showed accuracy within the acceptable range of FDA guidelines [28] (within 5%, Figure 3B). To evaluate matrix effects qualitatively, a postcolumn infusion system was employed to examine any reductions in the response of the target analytes in the EICs of blank DBS extracts following ACN‐based protein precipitation. As depicted in Figure 3C, stable ionization profiles were observed for the 11 drugs without notable enhancement or suppression zones during blank DBS analysis in LC–MS/MS. As a result, the chosen sample preparation method proved effective, yielding satisfactory recovery for all target drugs without causing interference in LC–MS/MS analysis. Importantly, untreated DBS papers were used in this study to reduce potential matrix effects, as chemically treated papers are typically more prone to interferences [27].

#### 3.2.2 Linearity, LOQ, and LOD

The method's linearity was evaluated by constructing calibration curves with at least six points within each drug's dynamic range. Calibration solutions were run alongside the samples during analysis, and the calibration curve measured in the same batch was used for quantification. Table S2 presents the equations and linear ranges for the quantification of the AEDs under the adjusted MS/MS settings. All drugs presented acceptable linearity across a wide concentration range, with linear regression correlation coefficients (*r* ^2^) greater than 0.998 from LOQ to limit of linearity (LOL) levels.

In microsampling matrices like DBSs, more precise LOQ criteria are required to reliably quantify targets measured at low concentrations. Therefore, instead of the conventional signal‐to‐noise method, the LOD and LOQ were determined following ICH guidelines, which provide a more rigorous and authoritative approach [22, 29]. The calculated LOD range was from 0.9 to 49.6 ng/mL, which, when compared to prior studies [30, 31, 32], demonstrates comparable performance.

#### 3.2.3 Accuracy and Precision

The accuracy and precision of the LC–MS/MS method were assessed by spiking drug‐free blood with AEDs at four levels spanning therapeutic and linear ranges. Spiked DBS samples were extracted and then analyzed utilizing the established method. Results from intra and interday assays (Table 1) demonstrate accuracy and precision across different concentration levels using six replicated samples. Both inter and intraday accuracy (% measured value/spiked value) and precision (% relative standard deviation) were within 6% for all target drugs, well within the 15% acceptable range in international guidelines [28, 29]. The consistent and quantitative recoveries of the target drugs and ISs validate the effectiveness of the extraction procedure for routine sample analysis.

**TABLE 1:** Accuracy (%) and precision (%) of the LC–MS/MS method for 11 AEDs spiked in DBSs by intra and interday assays (*n* = 6).

Drug	Gravimetric value (mg/kg)	Intraday		Interday	
		Accuracy (%)	Precision (RSD %)	Accuracy (%)	Precision (RSD %)
Vigabatrin	0.080	0.5	3.6	1.0	4.1
	0.11	0.4	6.9	−0.5	3.3
	0.29	1.0	5.1	0.6	2.5
	31	2.9	4.9	1.6	3.7
Levetiracetam	0.032	−0.3	3.5	−0.8	2.6
	1.2	−1.4	5.6	−0.4	3.9
	5.7	−5.1	4.4	1.1	4.8
	23	−1.8	6.0	−1.0	4.9
Pregabalin	0.041	3.3	5.2	2.0	3.0
	3.6	2.1	4.7	0.4	5.5
	7.8	0.8	5.1	2.8	3.4
	22	−1.0	5.5	4.5	3.8
Gabapentin	0.27	−1.3	5.2	−0.7	3.4
	1.8	−0.2	6.9	−3.2	3.9
	11	2.2	5.1	−0.6	5.4
	21	−4.9	3.6	−5.9	4.3
Lamotrigine	0.11	−3.6	6.2	−0.7	4.4
	1.2	−0.2	4.7	−2.3	3.9
	9.1	2.2	6.1	−3.3	5.5
	17	−1.1	4.9	−1.8	3.8
Lacosamide	0.11	2.2	4.6	−2.9	3.4
	2.1	−4.9	2.3	2.6	3.4
	13	0.7	3.3	1.5	5.9
	14	−1.8	5.2	−3.4	4.5
Zonisamide	0.11	2.7	4.1	2.7	3.6
	0.67	−0.4	3.4	0.4	3.3
	17	−3.8	4.6	−1.3	4.0
	33	−0.93	3.5	−2.4	3.4
Rufinamide	0.72	−3.3	3.8	2.4	3.7
	1.0	0.9	4.8	−0.6	3.2
	6.8	−2.2	6.3	0.5	3.7
	31	0.3	5.5	−5.1	6.8
Topiramate	0.063	5.1	2.5	−1.5	6.3
	2.9	−1.4	4.8	−2.7	4.3
	6.3	0.1	6.9	2.5	5.8
	13	−0.4	1.4	−2.9	2.7
Oxcarbazepine	0.061	−1.2	3.9	−2.2	5.3
	0.83	1.4	7.2	0.4	4.5
	11	0.7	3.3	−3.4	4.6
	39	−2.6	4.6	−1.6	4.2
Carbamazepine	0.050	1.4	4.6	2.2	3.9
	1.7	3.2	5.7	−2.0	1.2
	4.9	−3.1	6.0	5.4	2.6
	11	−3.5	6.6	−2.3	4.2

*Note:* Precision is calculated by standard deviation %; accuracy is calculated by relative error %; *n*: total number of replicates for each concentration.

### 3.3 Stability

Our previous paper [19] reported the stability of the standard solutions and the bench‐top stability, autosampler stability, and freeze–thaw stability of the AEDs; the current study focused on AED stability in DBSs, specifically examining both short‐ and long‐term stability. Generally, DBS samples are known to be advantageous for storage and transportation due to their dried state [33]. However, since the paper used for blood spot collection is typically not sterile and may be susceptible to contamination or other unforeseen factors, stability should be validated.

To assess the stability of the drugs in DBSs, we investigated different conditions covering short‐term stability (3 days and 1 week at room temperature) and long‐term stability (1 month at both room temperature and 4°C). In contrast to other sample types, DBS samples do not require freeze–thaw cycles, providing the benefit of excellent stability at ambient conditions. Assessing stability at room temperature is particularly important for shipping conditions and for making accurate comparisons with samples stored at 4°C [27]. These evaluation parameters are in accordance with international guidelines and recommendations [28].

Short‐term stability tests were conducted to evaluate sample storage and the potential for transportation to other clinical labs. The findings showed that all drugs in DBS samples retained stability for a minimum of 3 days at room temperature, with deviations not exceeding 5% (Table 2). Moreover, they demonstrated satisfactory stability at room temperature for up to 1 week (within 6%). Carbamazepine and oxcarbazepine, remarkably, showed better stability (within 6%) in DBS samples compared to serum form (> 17%) as observed in our previous study [25], particularly at room temperature for 3 days. This underscores the benefit of DBS samples in preserving drug stability compared to liquid biological samples.

**TABLE 2:** Stability tests of the 11 antiepileptic drugs spiked in DBSs at different storage conditions.

Drug	Gravimetric value (mg/kg)	Stability %^a^ (RSD %)^b^ (*n* = 6)			
		3 days at RT	1 week at RT	1 month at RT	1 month at 4°C
Vigabatrin	0.082	−3.2^a^ (5.0)^b^	−4.2^a^ (3.4)^b^	−6.5^a^ (4.1)^b^	−3.2^a^ (4.0)^b^
	0.19	−0.1 (2.1)	3.1 (5.3)	−5.1 (4.3)	1.2 (5.0)
	2.1	−1.0 (1.0)	−2.0 (4.4)	3.3 (0.3)	1.4 (3.0)
	31	−3.1 (4.2)	−3.1 (5.0)	2.2 (1.2)	1.4 (5.7)
Levetiracetam	0.043	−4.2 (4.4)	1.0 (0.3)	−9.1 (6.0)	4.3 (1.1)
	2.1	2.0 (5.4)	5.2 (1.1)	7.0 (1.1)	2.2 (6.2)
	6.9	1.0 (3.1)	0.4 (4.0)	−6.2 (3.0)	−1.4 (7.4)
	20	2.2 (2.3)	−4.0 (4.0)	1.4 (5.6)	0.3 (0.2)
Pregabalin	0.040	2.4 (1.0)	−3.3 (5.2)	7.5 (6.1)	2.6 (5.4)
	4.1	2.8 (4.1)	1.4 (1.4)	−7.8 (6.4)	1.1 (4.1)
	8.9	−1.9 (1.0)	0.1 (2.3)	5.0 (0.3)	1.0 (5.8)
	25	1.0 (1.3)	−2.4 (4.1)	−7.4 (2.1)	3.4 (2.0)
Gabapentin	0.24	1.0 (1.2)	5.1 (1.3)	5.2 (5.5)	1.2 (0.3)
	2.2	1.4 (2.0)	2.1 (5.2)	5.9 (5.9)	1.0 (9.2)
	9.7	2.0 (5.1)	0.4 (2.3)	4.1 (7.1)	0.4 (2.4)
	19	0.4 (4.3)	6.2 (1.4)	−7.0 (4.2)	3.3 (5.0)
Lamotrigine	0.014	1.4 (1.1)	1.3 (4.3)	4.5 (5.8)	1.4 (1.4)
	1.3	−2.2 (5.3)	0.4 (3.3)	−4.9 (1.6)	0.2 (8.4)
	2.8	−3.3 (1.4)	4.1 (2.1)	5.0 (0.3)	2.2 (6.2)
	19	−2.9 (0.1)	4.3 (2.3)	5.3 (0.1)	1.3 (6.4)
Lacosamide	0.022	0.41 (1.4)	−4.1 (4.1)	8.5 (3.2)	1.4 (4.2)
	3.1	0.25 (2.2)	1.1 (2.3)	8.1 (3.3)	4.3 (0.1)
	12	2.0 (3.0)	−4.2 (4.6)	6.6 (3.2)	3.2 (2.4)
	20	−1.1 (1.4)	0.7 (3.6)	5.1 (3.1)	4.2 (5.6)
Zonisamide	0.054	2.2 (1.1)	0.2 (4.0)	5.4 (2.3)	3.2 (2.2)
	0.70	−4.0 (5.1)	−4.1 (4.2)	3.0 (0.4)	2.3 (0.3)
	7.2	0.1 (4.1)	0.3 (4.1)	5.0 (7.3)	1.4 (1.1)
	35	−3.1 (4.4)	−5.4 (2.9)	4.4 (5.4)	0.3 (6.1)
Rufinamide	0.061	1.0 (5.1)	5.0 (1.1)	8.0 (0.3)	5.1 (2.4)
	1.1	−1.3 (3.1)	−0.3 (6.1)	4.2 (0.1)	4.2 (4.0)
	6.1	−4.1 (4.0)	−1.9 (0.7)	3.3 (7.4)	1.4 (4.2)
	36	−0.4 (2.3)	−2.2 (6.0)	5.2 (4.1)	1.1 (2.1)
Topiramate	0.064	4.1 (4.4)	−3.3 (4.1)	3.4 (5.4)	3.2 (3.3)
	2.9	3.0 (2.3)	2.4 (2.3)	6.3 (7.3)	2.2 (3.1)
	13	−2.0 (2.1)	0.2 (1.1)	8.1 (0.1)	4.7 (7.1)
	22	−2.0 (4.2)	0.1 (2.3)	4.4 (1.4)	0.8 (9.2)
Oxcarbazepine	0.050	1.3 (2.3)	0.3 (6.4)	9.3 (3.1)	5.2 (2.4)
	1.1	0.3 (3.2)	3.0 (5.3)	4.2 (0.4)	3.7 (4.1)
	16	−2.0 (4.1)	−4.0 (3.2)	7.3 (5.1)	4.4 (7.6)
	35	−2.1 (4.4)	−5.1 (2.4)	4.3 (6.7)	1.0 (7.0)
Carbamazepine	0.063	1.8 (2.4)	−2.3 (4.6)	7.2 (5.1)	0.2 (3.1)
	0.84	0.2 (1.9)	−0.4 (5.9)	7.4 (4.2)	1.1 (4.2)
	12	0.1 (1.0)	0.2 (1.0)	4.3 (0.4)	0.1 (6.3)
	19	1.0 (2.0)	−2.0 (2.3)	2.2 (9.1)	1.0 (7.4)

Abbreviation: RT: room temperature.

^a^ Stability (%) calculated as the measured value after storage/drug‐spiked value in serum.

^b^ Relative standard deviation (RSD, %).

For long‐term stability, the AEDs demonstrated strong stability (within 5%) when stored at 4°C for up to 1 month. Additionally, our study found that the AEDs in DBSs maintained stability for 30 days at room temperature (within 9%), offering valuable insights for blood sampling protocols for home‐based patients. These findings validate the method's reliability across different conditions and compliance with FDA standards for the accurate quantification of AEDs in DBS matrix [28].

### 3.4 Measurements From Disc Sampling

The method validation described above was conducted using whole blood spots prepared from 50 μL of blood in the laboratory. In clinical settings, though, controlling the volume of blood loaded onto each spot, such as with finger pricks, is challenging; as a result, a disc of consistent surface area is often used for analysis. The difficulty in ensuring accuracy with disc sampling arises from several factors. First, the volume of blood per unit area of the DBS can vary due to factors such as hematocrit, blood viscosity, temperature, and other potential influences [34, 35, 36, 37, 38]. Second, the distribution of target compounds within the DBS may vary depending on their physicochemical properties, such as solubility, hydrophobicity, molecular size, charge, partition coefficient, and interactions with blood components [39, 40]. These variations can lead to discrepancies in measurements, as different compounds may diffuse at different rates or associate with blood proteins, resulting in an uneven distribution within the spot.

The issue of variable blood volume per unit area in disc sampling has been widely studied and is a common source of bias not only for AEDs but also for the analysis of all compounds. In our previous report [41], we conducted a study to estimate the blood volume loaded onto a punched disc, finding that the gravimetric method proved to be the most accurate. This method involves measuring the weight of the paper before and after blood loading, as well as the weight of the whole spot and disc, and then calculating the blood volume by back‐calculating from these weight values. Using this method, we can calculate the blood volume per punched disc based on the blood used, although this value may vary with different levels of hematocrit or viscosity, which applies to all analytes, not just AEDs. In addition to the volume‐related issue, measurement bias arising from the heterogeneous distribution of AEDs within a DBS—due to factors such as differential diffusion or interactions with blood components—is also one of the main reasons why achieving accuracy in DBS measurements is challenging.

An experiment was conducted to determine whether the measured values from serum, whole blood, whole DBSs, central‐punched discs, and peripheral‐punched discs matched the concentrations of spiked AEDs. The results are shown in Table 3, which indicates the measurement values of the AEDs in the five above sources with known amounts of AEDs, exhibiting that their accuracies did not exceed 5% in all AEDs. No significant differences (< 5%) were observed between central‐punched and peripheral‐punched discs, suggesting that the target AEDs were relatively evenly distributed within the DBS.

**TABLE 3:** Measurement results (mean ± SD, *n* = 4) of the 11 AEDs from disc sampling, whole spot sampling, whole blood, and serum.

Drug	Gravimetric value (mg/kg)	DBS			Blood	Serum
		Punch‐in (center)	Punch‐out (periphery)	Whole		
Vigabatrin	9.90	10.1 ± 0.3	10.1 ± 0.3	10.5 ± 0.6	9.52 ± 0.42	10.0 ± 0.6
Levetiracetam	21.3	21.6 ± 1.1	21.3 ± 0.3	21.1 ± 1.2	21.1 ± 0.5	22.2 ± 1.1
Pregabalin	7.71	7.41 ± 0.63	7.71 ± 0.56	7.44 ± 0.41	7.81 ± 0.23	7.51 ± 0.50
Gabapentin	11.0	11.0 ± 0.3	11.2 ± 0.4	11.2 ± 0.7	11.2 ± 0.4	11.5 ± 0.6
Lamotrigine	12.3	12.2 ± 0.4	12.6 ± 0.3	12.0 ± 0.2	12.0 ± 0.4	12.3 ± 0.2
Lacosamide	13.0	13.1 ± 0.1	13.5 ± 1.2	13.0 ± 0.5	13.2 ± 0.9	13.2 ± 0.6
Zonisamide	22.1	22.1 ± 0.3	20.6 ± 2.5	21.1 ± 1.8	21.5 ± 1.1	22.1 ± 0.5
Rufinamide	15.1	15.2 ± 0.7	15.1 ± 0.2	16.6 ± 1.7	15.1 ± 0.6	15.0 ± 0.6
Topiramate	25.1	24.1 ± 1.1	24.2 ± 1.8	25.4 ± 0.9	24.2 ± 1.4	24.1 ± 1.3
Oxcarbazepine	30.7	30.2 ± 1.1	30.1 ± 1.3	30.5 ± 0.8	31.2 ± 1.2	31.1 ± 1.4
Carbamazepine	10.5	10.4 ± 0.2	10.4 ± 0.3	10.2 ± 0.4	10.4 ± 0.6	10.5 ± 0.2

As a result, the accuracies of all measurements remained within 5% when the blood volume within the discs was accurately quantified using the gravimetric method, indicating that the small differences between central‐punched and peripheral‐punched discs are likely caused by random variability rather than a location‐based systematic bias. However, the variation observed in actual patient samples with different hematocrit levels must also be considered. To predict the maximum error when combining measurement variation and hematocrit‐related bias, we used the standard method of uncertainty propagation, which involves squaring each factor, summing them, and then taking the square root. A previous study [42] modeled the area differences resulting from hematocrit values in the 20%–60% range, predicting a bias of approximately 13%–14%. In this case, even when combining the hematocrit‐induced bias (13.5%) with our random variation (5%), the combined error did not exceed the acceptance criteria (< 15%). For typical adult blood with hematocrit values between 35% and 50%, calculated variations in blood area were maximally 10%, according to various studies [38, 43]. In such cases, even if the measurement‐related random variation reaches 10%, the combined variation remains within the 15% acceptance criteria. In other words, our method ensures sufficient accuracy to meet the acceptance criteria even when accounting for hematocrit‐induced bias, making it feasible for future measurements. Yet, it is important to note that this is an estimate based on existing studies; further data accumulation from a wide range of patient samples will be necessary for more accurate conclusions.

## 4 Conclusion

Considering the growing use of microsampling techniques, such as DBS sampling, beyond traditional serum‐based analysis methods, this study conducted a series of evaluations to validate the efficacy of a DBS‐based AED analysis method. The extraction efficiency of AEDs from DBSs was evaluated using organic solvents ACN and MeOH, and it was confirmed that ACN enabled the efficient extraction of all AEDs. LC–MS/MS analysis of the extracted samples demonstrated good selectivity for the AEDs, with no matrix effects or carryover. The accuracy and precision were within 6% for all AEDs across four concentration levels in intra and interday assays, showing acceptable ranges as recommended by FDA guidelines. Additionally, stability testing of the AEDs in DBS samples confirmed that stability could be maintained for up to 1 month, both at room temperature and at 4°C, with carbamazepine and oxcarbazepine exhibiting superior stability in DBS samples compared to serum samples. Finally, analysis of punched discs from DBS samples revealed uniform results with no bias between central and peripheral samples, indicating that measurement bias related to different punch locations within the whole spot is negligible. This suggests that the DBS‐based AED analysis method provides sufficient reliability to serve as an alternative to traditional serum‐based methods, provided that the common issue of volume variation per unit area is overcome. In sum, this study underscores the potential of DBS‐based AED analysis as a noninvasive and convenient solution for TDM.

## Author Contributions

**Mariam M. Abady:** methodology, writing – original draft, investigation, validation. **Ji‐Seon Jeong:** investigation, supervision. **Ha‐Jeong Kwon:** conceptualization, writing – review and editing, resources, funding acquisition.

## Conflicts of Interest

The authors declare no conflicts of interest.

### Peer Review

The peer review history for this article is available at https://www.webofscience.com/api/gateway/wos/peer‐review/10.1002/rcm.10064.

## Supporting information


**Table S1.** Enhanced MS/MS parameters for the extracted ion chromatograms (EICs) of AED target analytes and their isotope internal standards in positive ESI mode.
**Table S2.** Clinical therapeutic range and linearity details, including limits of linearity, linear equation, correlation coefficient of the calibration curve, and lower limits of detection and quantification for the target drugs analyzed by LC–MS/MS.
**Figure S1.** Carryover of AEDs spiked in DBS samples. Carryover was measured by injecting a blank DBS sample after the highest level of calibrator. Error bars represent the standard deviation for three replicates (*n* = 3 samples).

## Data Availability

The data that support the findings of this study are available from the corresponding author upon reasonable request.
