# Supplementary material for: Dried Blood Spot Analysis for Simultaneous Quantification of Antiepileptic Drugs Using Liquid Chromatography–Tandem Mass Spectrometry
Source: Rapid Commun Mass Spectrom. 2025 May 12;39(16):e10064. doi: 10.1002/rcm.10064 (PMC12127245; doi:10.1002/rcm.10064)
Supplement: Supplementary file 1 — Table S1. Enhanced MS/MS parameters for the extracted ion chromatograms (EICs) of AED target analytes and their isotope internal standards in positive ESI mode. Table S2. Clinical therapeutic range and linearity details, including limits of linearity, linear equation, correlation coefficient of the calibration curve, and lower limits of detection and quantification for the target drugs analyzed by LC–MS/MS. Figure S1. Carryover of AEDs spiked in DBS samples. Carryover was measured by injecting a blank DBS sample after the highest level of calibrator. Error bars represent the standard deviation for three replicates (n = 3 samples). [file RCM-39-e10064-s001.docx]

Supplementary material

**Dried blood spot analysis for simultaneous quantification of eleven antiepileptic drugs using liquid chromatography–tandem mass** **spectrometry**

Mariam M. Abady^1,2,3^, Ji-Seon Jeong^1,2^, Ha-Jeong Kwon^1*^

*^1^Organic Metrology Group, Division of Chemical and Material Metrology, Korea Research Institute of Standards and Science, Yuseong-gu, Daejeon 34113, Republic of Korea*

*^2^Department of Bio-Analytical Science, University of Science and Technology, 217 Gajeong-ro, Yuseong-gu, Daejeon 34113, Republic of Korea*

*^3^Department of Nutrition and Food Science, National Research Centre, Dokki, Cairo 12622, Egypt*

∗ Correspondence should be addressed to Dr. Ha-Jeong Kwon

E-mail: [hajeong.kwon@kriss.re.kr](mailto:hajeong.kwon@kriss.re.kr)

Tel: 82-42-868-5833

**Supplementary text**

**Text S1.** **Sample preparation for comparison of extraction efficiency**

Dried blood spot (DBS) samples were prepared by adding 150 µL of the antiepileptic drugs (AEDs) standard mixture (level 3; see Section 2.3 in the main text) in 10 mL of human drug-free whole blood, which was gently homogenized for 2 h at 4 °C. Next, 50 μL of the mixture was dispensed onto filter paper and allowed to dry overnight at 4 °C in the dark. Deionized water (DW, 300 µL) was then added to the DBS sample volumetrically, followed by gentle shaking for 1 h at room temperature. The spiked DBS was purified by adding 700 µL of miscible organic solvent (acetonitrile (ACN), methanol, and their mixtures in ratios of 2:1, 1:1, and 1:2) and shaken well for 1 h at room temperature. After centrifugation for 20 min at 16200*g*, the supernatant was transferred to a 2 ml tube and evaporated to dryness in a vacuum evaporator at 55 ^o^C. The residue was subsequently reconstituted in 50 µL of methanol, 50 µL of distilled water, and 50 µL of internal standard mixture to a total volume of 150 µL and filtered using a PVDF microspin filter device (pore size 0.22 μm). Finally, the samples were injected into LC-MS/MS for comparison of the extraction efficiency.

**Text S2.** **Chromatographic separation conditions**

Chromatographic separations were carried out in a Zorbax SB-Aq reversed-phase column (150 mm length, 2.1 mm internal diameter, 3.5 μm particle size) paired with a Zorbax SB-Aq Guard pre-column (5 mm length, 2.1 mm internal diameter, 3.5 μm particle size) from Santa Clara, CA, USA. The column temperature was consistently kept at 30 °C, with the flow rate set to 0.2 mL/min and a sample injection volume of 5 μL. The samples were kept in the autosampler at 4 °C, and the injection needle was cleaned using 5% ACN after each injection to reduce potential carryover. The mobile phases were prepared as follows: mobile phase A was composed of 2 mM ammonium formate and 0.1 (v/v) % formic acid in DW, while mobile phase B was composed of 0.1 (v/v) % formic acid in ACN. The gradient program began with 5% B for 3 min, then increased linearly to 20% B for 2 min, and holding at 20% B for additional 2 min. This was followed by a linear increase to 80% B over 3 min, a linear decrease back to 5% B for 1 min, and finally a 2 min re-equilibration at the initial condition of 5% B to prepare the column for the next analysis.

**Table S1. Enhanced MS/MS parameters for the extracted ion chromatograms (EICs) of AED target analytes and their isotope internal standards in positive ESI mode**

| Drug | Molecular formula | LC retention time (min) | Precursor ion  (m/z) | Product ion  (m/z) | CE^c^  (V) | DP^d^ (V) |
| --- | --- | --- | --- | --- | --- | --- |
| Vigabatrin | C_6_H_11_NO_2_ | 2.3 | 130.1 | 113.1^a^  116.1^b^ | 10 | 30 |
| Levetiracetam | C_8_H_14_N_2_O_2_ | 4.4 | 171.1 | 154.1^a^  126.1^b^ | 10 | 40 |
| Pregabalin | C_8_H_17_NO_2_ | 4.9 | 160.1 | 142.1^a^  124.1^b^ | 18 | 40 |
| Gabapentin | C_9_H_18_Cl NO_2_ | 5.1 | 172.1 | 154.1^a^  137.1^b^ | 20 | 54 |
| Lamotrigine | C_9_H_7_Cl_2_N_5_ | 8.2 | 256.0 | 210.9^a^  186.9^b^ | 30 | 65 |
| Lacosamide | C_13_H_18_N_2_O_3_ | 8.5 | 251.1 | 108.1^a^  219.1^b^ | 25 | 60 |
| Zonisamide | C_8_H_8_N_2_O_3_S | 8.6 | 213.0 | 132.0^a^  149.0^b^ | 25 | 40 |
| Rufinamide | C_10_H_8_F_2_N_4_O | 9.5 | 239.1 | 127.0^a^  222.0^b^ | 25 | 60 |
| Topiramate | C_12_H_21_NO_8_S | 10.4 | 340.3 | 276.0^a^  224.2^b^ | 15 | 40 |
| Oxcarbazepine | C_15_H_12_N_2_O_2_ | 10.8 | 253.1 | 180.1^a^  208.1^b^ | 40 | 60 |
| Carbamazepine | C_15_H_12_N_2_O | 11.1 | 237.1 | 194.1^a^  179.1^b^ | 35 | 60 |
| ACHC | C_7_H_13_NO_3_ | 1.8 | 144.1 | 126.1^a^ | 25 | 60 |
| Levetiracetam-D_6_ | C_8_D_6_H_8_N_2_O_2_ | 4.4 | 177.1 | 160.1^a^ | 10 | 40 |
| Gabapentin-^13^C_3_ | C_6_^13^C_3_H_17_NO_2_ | 5.1 | 175.1 | 157.1^a^ | 20 | 54 |
| Lamotrigine ^13^C,^15^N_4_ | ^13^CC_8_H_7_Cl_2_^15^N_4_N | 8.2 | 261.0 | 213.9^a^ | 30 | 65 |
| Lacosamide-D_3_ | C_13_ D_3_H_15_N_2_O_3_ | 8.5 | 254.2 | 108.1^a^ | 25 | 60 |
| Oxcarbazepine-^13^C | ^13^CC_14_H_12_N_2_O_2_ | 10.8 | 259.1 | 186.1^a^ | 40 | 60 |

^a^ quantifier ion; ^b^ qualifier ion; ^c^ CE: collision energy; ^d^ DP: declustering potential; ACHC: 4- amino cyclohexane carboxylic acid

The following common MS/MS parameters were used for all analyses: ion source temperature = 500 °C, ion spray voltage floating = 5500 V, curtain gas pressure (nitrogen) = 30 psi, GS1 (sheath gas) = 50 psi, and GS2 (heating gas) = 50 psi.

**Table S2. Clinical therapeutic range and linearity details, including limits of linearity, linear equation, correlation coefficient of the calibration curve, and lower limits of detection and quantification for the target drugs analyzed by LC-MS/MS**

| Drug | Therapeutic range (µg/mL) |  |  |  | Linearity range  (ng/mL) | Linear equation | r^2^ | LOD  (ng/mL) | LOQ  (ng/mL) |
| --- | --- | --- | --- | --- | --- | --- | --- | --- | --- |
| Vigabatrin | 0.8–36 |  |  |  | 5–25000 | y = 3.2419x + 0.1157 | 0.9991 | 0.9 | 4.8 |
| Levetiracetam | 5–40 |  |  |  | 1–25000 | y = 1.0025x + 0.1606 | 0.9985 | 0.3 | 0.9 |
| Pregabalin | 2–8 |  |  |  | 5–25000 | y = 1.8098x - 0.0573 | 0.9999 | 1.4 | 4.5 |
| Gabapentin | 2–20 |  |  |  | 14–10000 | y = 1.0240x + 0.0249 | 0.9999 | 4.6 | 13.9 |
| Lamotrigine | 2–20 |  |  |  | 3–10000 | y = 1.0466x + 0.0092 | 0.9981 | 1 | 3 |
| Lacosamide | 10–20 |  |  |  | 5–10000 | y = 0.9805x + 0.0348 | 0.9995 | 1.4 | 4.4 |
| Zonisamide | 10–40 |  |  |  | 25–25000 | y = 0.0876x + 0.0920 | 0.9977 | 8.1 | 25 |
| Rufinamide | 10–40 |  |  |  | 2.3–25000 | y = 1.0725x + 0.0169 | 0.9988 | 0.76 | 2.3 |
| Topiramate | 5–20 |  |  |  | 4.5–10000 | y = 1.1904x + 0.0452 | 0.9989 | 1 | 4.5 |
| Oxcarbazepine | 10–40 |  |  |  | 2.3–25000 | y = 1.469x + 0.4099 | 0.9988 | 0.75 | 2.3 |
| Carbamazepine | 4–12 |  |  |  | 1.4–25000 | y = 1.5308x + 0.1442 | 0.9990 | 0.46 | 1.4 |

**Supplementary Figures**

**Fig. S1**. Carryover of AEDs spiked in DBS samples. Carryover was measured by injecting a blank DBS sample after the highest level of calibrator. Error bars represent the standard deviation for three replicates (n = 3 samples).
